# Supplementary material for: Microencapsulates of Blue Maize Polyphenolics as a Promising Ingredient in the Food and Pharmaceutical Industry: Characterization, Antioxidant Properties, and In Vitro-Simulated Digestion
Source: Foods. 2023 Apr 30;12(9):1870. doi: 10.3390/foods12091870 (PMC10178619; doi:10.3390/foods12091870)
Supplement: Supplementary file 1 [file foods-12-01870-s001.zip › foods-2345869-supplementary.pdf]

blue popping maize

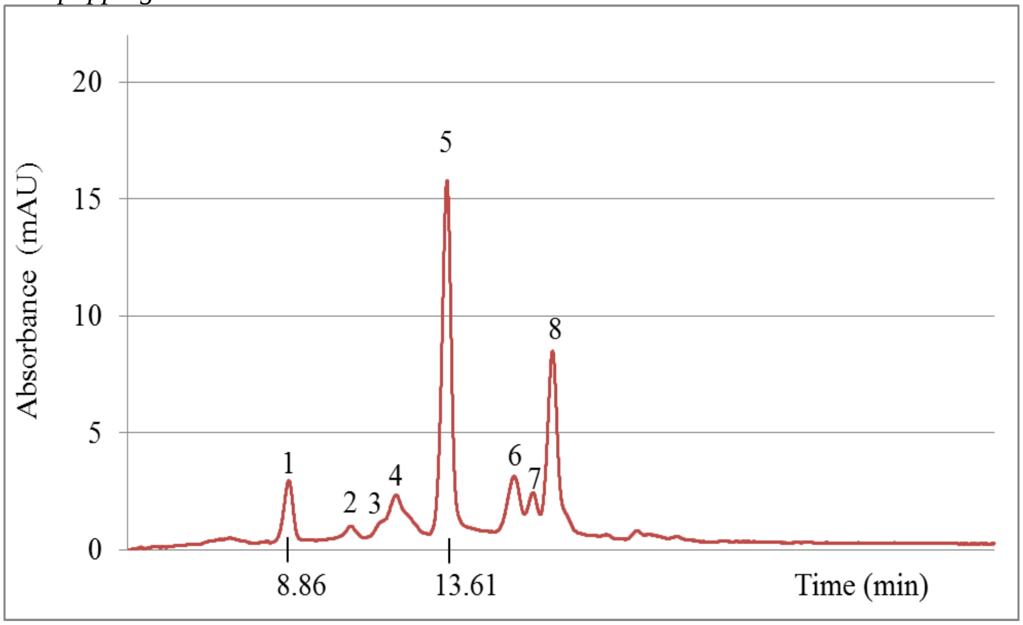

Figure S1. Chromatogram at 530 nm corresponding to blue popping maize. The peak numbers show the major anthocyanins that are identified in Table S1.

Table S1. Anthocyanins composition in grains of blue popping maize. Chromatographic characteristics of peaks in Figure S1.

Peak	t_R (min)	Area (%) (535)	$[M^+]$ (m/z)	MS/MS (m/z)	Identity
1	8.87	6.68	449	287	Cyanidin 3-glucoside
2	10.73	1.40	433	271	Pelargonidin 3-glucoside
3	11.70	1.74	463	301	Peonidin 3-glucoside
4	12.08	6.14	535	287	Cyanidin 3-(3'-malonylglucoside)[1]
5	13.61	41.01	535	287	Cyanidin 3-(6'-malonylglucoside)[1]
6	15.63	9.73	621	438/287	Cyanidin 3-(dimalonyl-β-glucoside)[2]
	15.71	trace	519	303	Pelargonidin 3-(6'-malonylglucoside)
7	16.19	5.00	621	438/287	Cyanidin 3-(dimalonyl-β-glucoside)[2]
	16.60	trace	549	301	Peonidin 3-(6'-malonylglucoside)
8	16.77	23.06	621	438/287	Cyanidin 3-(dimalonyl-β-glucoside)[2]

[1-2]Compounds with identical m/z ratio within superscript.
